# Ophthalmic Disorders in Adult Lymphoma Patients in Africa

**DOI:** 10.4103/0974-9233.58420

**Published:** 2009

**Authors:** Afekhide E Omoti, Caroline E Omoti, Rita O Momoh

**Affiliations:** Department of Ophthalmology, University of Benin Teaching Hospital, PMB 1111, Benin City, Edo State, Nigeria; 1 Department of Hematology, University of Benin Teaching Hospital, PMB 1111, Benin City, Edo State, Nigeria

**Keywords:** Lymphoma, Ophthalmic Disorders, Proptosis

## Abstract

**Context:**

Ocular manifestations of lymphoma are rare events. Most reports of ocular involvement in lymphoma are case reports or reports of a few patients.

**Aims:**

To determine the ophthalmic disorders in adult, African, lymphoma patients.

**Settings and Design:**

A prospective study of ocular disorders in adult patients with lymphoma was conducted at the University of Benin Teaching Hospital, Benin City, Nigeria, between July 2004 and June 2007.

**Materials and Methods:**

The patients were interviewed and examined by the authors and the ocular findings recorded.

**Statistical Analysis:**

Data was analyzed on computer with the aid of the Instat GraghPad™ v2.05a statistical package software. The mean, standard deviation, Mann-Whitney U-statistic and P value were calculated.

**Results:**

A total of 111 patients with hematological malignancies were seen over a period of three years of which 62 (55.85%) had lymphomas. Of these, 51(82.3%) were non-Hodgkin's lymphoma and 11(17.7%) were Hodgkin's lymphoma. Ocular disorders occurred in 16 patients (31.4%) with non-Hodgkin's lymphoma and none of the patients with Hodgkin's lymphoma (Mann-Whitney U-statistic is equal to 7.500, U' is equal to161.50, P< 0.0001). The ocular disorders due to non-Hodgkin's lymphoma were seen as – proptosis in six patients (11.8%), retinopathies in three (5.9%), conjunctival infiltration in three (5.9%), optic atrophy in two (3.9%), keratoconjunctivitis in one (two per cent), desquamating nodular lid lesions in one (two per cent), papilloedema in one (two per cent), and upper lid mass in one (two per cent). Four patients (6.5%) had monocular blindness.

**Conclusions:**

Ophthalmic disorders are relatively common in non-Hodgkin's lymphoma. Ophthalmic evaluation is needed in these patients for early identification and treatment of potentially blinding conditions.

## INTRODUCTION

Lymphomas are a heterogeneous group of malignancies of B cells, T cells and rarely natural killer (NK) cells that usually originate in the lymph nodes but may originate in any organ of the body.^1^ Non-Hodgkin's primary central nervous system lymphoma, reactive lymphoid hyperplasia, and systemic non-Hodgkin's lymphoma have been reported to affect all the intraocular and orbital structures.^2^–^7^  Cases of mycosis fungoides and Sezary syndrome with ocular involvement have also been reported.^8^,^9^ Hodgkin's lymphoma has also been reported to affect ocular structures.^10^

Most reports of ocular involvement in lymphoma are case reports or reports of a few patients.^11^  This may reflect its relative rarity. Since an indeterminate number of unreported and isolated cases have occurred, meaningful data on incidence and prevalence is not available. In general, the primary non-Hodgkin's lymphoma of the CNS is rare, accounting for one per cent of all non-Hodgkin's lymphomas, one per cent of intracranial tumors and less than one per cent of all intraocular tumors.^2^,^12^

Intraocular lymphoma is probably the most elusive intraocular tumor to diagnose.^11^  Diagnosis can be difficult and is frequently delayed as the clinical condition can mimic a number of other ocular conditions.^2^,^7^  Further, ocular manifestations of lymphoma are generally rare events.^11^,^13^  Late presentation in advanced stages of disease is common in African patients.^14^  The pattern of ocular disease in African patients with hematological malignancies may thus be different from that reported in studies from more advanced countries. This study was designed to identify the common ocular manifestations of lymphoma in African eyes.

## MATERIALS AND METHODS

All new adult patients with a diagnosis of any of the lymphomas attending haemato-oncology clinics or admitted into the medical wards of the University of Benin Teaching Hospital, Benin City, Nigeria, between July 2004 and June 2007 were evaluated in the Eye clinic of the hospital. Diagnosis was established based on tissue samples studied by histological examination of a surgical biopsy from an accessible lymph node site or orbito-ocular tissue by the Morbid Anatomy department of the same institution. They were classified as Hodgkin's and non-Hodgkin's lymphoma.

Exclusion criteria included all patients who had received chemotherapy, patients on follow-up and children below 17 years. All new adult patients were interviewed by the authors using a standard questionnaire and examined in the eye clinic. The visual acuity was determined using the Snellen's chart or illiterate E chart held at six meters from the patient. When the patient could not read the chart, the ability to count fingers at varying distances, to perceive hand movements or light was determined and recorded. Near vision was tested using the Jaeger's reading chart. The eyes were examined using a pen torch, Haag Streit slit-lamp biomicroscope and direct ophthalmoscope. Fluorescein staining of corneal lesions was done whenever indicated. The intraocular pressure was measured using the Goldmann applanation tonometer mounted on the Haag Streit slit-lamp. Visual field analysis was done using the Kowa automatic visual field plotter. Indirect ophthalmoscopy was done after dilatation of the pupils with 2.5% phenylephrine hydrochloride and 0.5% tropicamide.

Visual acuity was also measured with a pinhole. Those who showed improvement in vision with pinhole and those who complained of difficulty with reading were refracted and appropriate lenses were prescribed. The best corrected visual acuity was used to define blindness. Blindness was defined using the World Health Organization International classification of diseases (10^ th^ revision, 1994) definition which states that blindness is best corrected vision less than 3/60 or inability to count fingers at three meters. Visual impairment was defined as best corrected vision less than 6/18. Ethical approval for this study was obtained from the Ethical Committee of the University of Benin Teaching Hospital, Benin City, Nigeria.

Data obtained from this study was analyzed on computer with the aid of the Instat GraghPad™ v2.05a statistical package software. An initial frequency count and percentages were obtained for all the data. The range, mean, median, standard deviation (SD), standard error of mean (SEM) and 95% confidence intervals (CI) were determined for ages of the patients. Relevant data was displayed in a tabular form. Statistical significance was calculated using the Mann-Whitney U-statistic and a *P* value of less than 0.05 was considered significant.

## RESULTS

A total of 111 consecutive new adult patients with various hematological malignancies were seen during the period of study. They included 62 cases of malignant lymphomas (55.9%), 37 cases of leukemia (33.3%), nine cases of myelomatosis (8.1%), two cases of polycythemia rubra vera (1.8%) and one case of idiopathic myelofibrosis (0.9%). Of the 62 cases of lymphoma, 51(82.3%) were non-Hodgkin's lymphoma and 11(17.7%) were Hodgkin's lymphoma. None of the patients had received treatment elsewhere before presentation.

There were 41 males (66.1%) and 21 females (33.9%) giving a male to female ratio of 1.95:1. The age range was 26 to 75 years with a mean of 49.36 years (SD plus/minus 15.6). The SEM was 4.73 and the median age was 52 years. The 95% CI was 38.82–59.90 years.

The most common ocular disorders found on examination of the patients were refractive errors/presbyopia, proptosis, cataract and retinopathies [Table 1].

**Table 1:** Ocular disorders in lymphoma patients

Ocular disorders	Hodgkin's No. (%)	Non-Hodgkin's No. (%)
Refractive error/presbyopia	2(18.2)	10(19.6)
*Proptosis	0(0)	6(11.8)
Cataract	0(0)	4(7.8)
*Retinopathies	0(0)	3(5.9)
*Optic atrophy	0(0)	2(3.9)
Allergic conjunctivitis	1(9.1)	3(5.9)
Age related macula degeneration	0(0)	2(3.9)
*Conjunctival infiltration	0(0)	3(5.9)
Pterygium	0(0)	2(3.9)
*Keratoconjunctivitis	0(0)	1(2.0)
*Erythematous desquamating skin	0(0)	1(2.0)
*Papilloedema	0(0)	1(2.0)
* Upper lid mass	0(0)	1(2.0)

* Mann whitney U-statistic = 7.500, U = 161.50, *P* < 0.0001; *Ocular disorders due to lymphoma

Some patients had more than one ocular disorder. The ocular disorders directly related to lymphoma included proptosis, retinopathies, optic atrophy, conjunctival infiltration by malignant tissue, lid mass, the erythematous desquamating skin lesions, keratoconjunctivitis and papilloedema [Table 1]. These ocular disorders occurred in 16 patients (31.4%) with non-Hodgkin's lymphoma and none of the patients with Hodgkin's lymphoma. All the patients with proptosis and the upper lid mass presented first to the eye clinic and biopsy of ocular or lid tissue revealed non-Hodgkin's lymphoma. In five cases, proptosis was unilateral and in one case it was bilateral. Retinopathy included cases of retinal hemorrhages and vasculitis. The retinopathy was presumed to be due to the lymphoma. The patient who presented with a massive upper lid mass involving the forehead initially presented a diagnostic difficulty until biopsy was performed which revealed non-Hodgkin's lymphoma. The erythematous desquamating skin lesions involving the lids and keratoconjunctivitis occurred in a case of mycosis fungoides.

No patient with lymphoma was blind at presentation but seven cases (11.3%) were visually impaired [Table 2].

**Table 2:** Visual acuities in eyes of lymphoma patients

Visual acuity	Better eye (%)	Worse eye (%)
6/4-6/9	36 (58.1)	24 (38.7)
6/12-6/18	19 (30.6)	22 (35.5)
6/24-CF 3 metres	7 (11.3)	12 (19.3)
<CF3m-NLP	0 (0)	4 (6.5)
Total	62 (100)	62 (100)

* CF = Counting fingers; NLP = No light perception

Four patients (6.5%) had monocular blindness. The causes of monocular blindness were cataract (one case), proptosis (one case) and optic neuropathy (two cases).

## DISCUSSION

Late presentation of patients with hematological malignancies is a problem in Nigeria, and this has been documented in previous studies from the same institution.^14^,^15^  Ocular disorders may be the presenting features or may complicate already diagnosed lymphoma. 3,4,6,11  Most of the primary intraocular lymphomas are malignant B-cells while intraocular T-cell lymphomas are uncommon.^16^  Several symptoms and ocular disorders identified on ocular examination were not related to the lymphoma in this report. These included refractive errors, presbyopia, cataract, allergic conjunctivitis, age related macular degeneration and pterygium. These are conditions which are common in the general population. The conditions directly related to lymphoma included cases of proptosis, retinopathy, optic atrophy, conjunctival infiltration, erythematous desquamating skin lesions, papilloedema and upper lid mass. The total number of cases with ocular involvement due to the malignancy was 16 representing 31.4% of cases of non-Hodgkin's lymphoma and 25.8% of all cases of lymphoma. This Figure is relatively high since other studies from advanced countries have reported that ocular disorders in lymphoma are relatively rare events.^11^,^13^  Late presentation, in advanced stages of the disease, may have contributed to this relatively high incidence. The absence of ocular complications in a few cases of Hodgkin's disease is in keeping with the relative rarity in other studies.^11^,^13^

The lymphomas were grouped simply into Hodgkin's disease and non-Hodgkin's lymphomas because of the lack of adequate facilities to further sub classify them and because of the small numbers available. Using the Revised European-American Lymphoma Classification (REAL),^17^  most of the patients with non-Hodgkin's lymphoma belonged to the diffuse large B-cell lymphoma and follicle center-cell lymphoma, follicular subtypes while the majority of patients with Hodgkin's disease belonged to the lymphocyte depletion and mixed cellularity sub types.

There were more males in this study. This is in agreement with studies that show a male preponderance in lymphoma.^18^  However, this may be a manifestation of the increased tendency of males to seek health services in sub-Saharan Africa. The most common specific ocular manifestation of lymphoma in this report was proptosis. It is due mainly to infiltration of the orbital tissues by malignant cells resulting in displacement of the eyeball forwards.^11^  All the six cases of proptosis presented first to the eye clinic. In addition, the patient with an upper lid mass presented with a massive tumor involving the upper lid and forehead. This patient presented first to the eye clinic and was a diagnostic difficulty until biopsy revealed that it was a case of non-Hodgkin's lymphoma. Thus a total of 7 cases of lymphoma (11.3%) presented first to the eye clinic before the definitive diagnosis was made.

The ocular changes result either from a direct effect of metastatic neoplastic infiltration or compression or by circulating antibodies involving paraneoplastic retinal degeneration, or simply from increased susceptibility to infections as a result of immunosuppression that these patients undergo.^11^  Infiltration and effect of circulating antibodies may be responsible for the retinopathies while infiltration and compression may be responsible for optic atrophy and papilloedema. Suppression of immunity may have contributed to one case of severe keratoconjunctivitis which occurred in a case of non-Hodgkins lymphoma.^11^

The uniocular blinding conditions included cataract, proptosis and optic neuropathy. In addition, retinopathies, age related macula degeneration and uncorrected refractive errors were responsible for visual impairment. All these conditions are either treatable or preventable but early diagnosis is required in most of the cases to prevent blindness.

In conclusion, ocular morbidity is relatively common in patients with lymphoma in Benin City, Nigeria. Patients with lymphoma may present first to the eye clinic with proptosis or a mass lesion in the periocular tissues. Several of these are potentially blinding conditions. Early ophthalmic evaluation is recommended to identify and treat those conditions.

## ACKNOWLEDGMENT

We wish to acknowledge the assistance of the resident doctors in the Department of Hematology and Ophthalmology of the University of Benin Teaching Hospital, Benin City, Nigeria in data collection.
